# High expression of Lin28 is associated with tumour aggressiveness and poor prognosis of patients in oesophagus cancer

**DOI:** 10.1038/bjc.2012.90

**Published:** 2012-03-20

**Authors:** R Hamano, H Miyata, M Yamasaki, K Sugimura, K Tanaka, Y Kurokawa, K Nakajima, S Takiguchi, Y Fujiwara, M Mori, Y Doki

**Affiliations:** 1 Department of Gastroenterological Surgery, Osaka University Graduate School of Medicine, Suita, Yamadaoka 2-2, Osaka 565-0871, Japan

**Keywords:** Lin28, Lin28B, oesophageal cancer, let-7

## Abstract

**Background:**

Lin28 is a negative regulator of the tumour suppressor microRNA, let-7, suggesting its role in tumourigenesis. However, the clinical significance of Lin28 expression in oesophageal cancer has not been elucidated.

**Methods:**

Lin28 and Lin28B expression was examined by immunohistochemistry in 161 tissues from patients with oesophageal cancer who had undergone curative surgery. The relationship between the expressions of Lin28 and Lin28B and various clinicopathological factors was examined. *In vitro* assays were conducted to determine the role of Lin28 in aggressiveness of oesophageal cancers using oesophageal cancer cell line.

**Results:**

Lin28 and Lin28B were overexpressed in oesophageal cancer cells compared with non-cancerous epithelial cells, especially in the invasive front. High expression of Lin28 and Lin28B correlated significantly with lymph node metastasis and poor prognosis. High expression of Lin28B expression correlated significantly with low expression of let-7. Multivariate analysis also identified Lin28B expression as an independent prognostic factor. *In vitro* assays showed that the proliferative and invasive activities were significantly reduced in Lin28B-knockdown cells, compared with control cells.

**Conclusion:**

High expression of Lin28 is associated with poor prognosis and high tumour aggressiveness in oesophageal cancer and these effects are mediated through increased proliferation and invasiveness of oesophageal cancer cells.

MicroRNAs (miRNAs) are a class of small nonprotein-coding RNAs that act by endogenous interference. They bind to the 3′ untranslated region of the target mRNAs, leading to translational repression or reduced stability of the mRNA (Bartel, 2004). MicroRNAs are known to have important roles in various biological processes, such as cell differentiation, cell proliferation, apoptosis, and metabolism. MicroRNAs also have emerged as central regulators of cancer (Calin and Croce, 2006). Their aberrant expression in many tumours indicates that they could function as tumour suppressors or oncogenes. Moreover, there is increasing evidence that miRNA expression is potentially useful biomarker for the diagnosis, prognosis and tailoring of therapy for patients with various cancers, but the mechanism involved in the regulation of miRNA expression is still uncertain. Recent studies have indicated that miRNA biogenesis can be regulated posttranscriptionally by *trans*-acting factors (Lee *et al*, 2003; Denli *et al*, 2004). It is reported that the biogenesis of let-7 family members, which seem to act as tumour suppressor miRNAs, is negatively regulated posttranscriptionally by Lin28 in embryonic stem cells and certain cancer cell lines (Heo *et al*, 2008; Newman *et al*, 2008; Piskounova *et al*, 2008; Viswanathan *et al*, 2008; Hagan *et al*, 2009).

Lin28 is a RNA-binding protein originally identified as a key regulator of developmental timing in *Caenorhabditis elegans* (Moss *et al*, 1997). Lin28 is a conserved cytoplasmic protein but is exported to the nucleus where it seems to regulate the translation or stability of mRNAs by localising them to P-bodies (Balzer and Moss, 2007). In mammals, Lin28 is widely expressed in embryonic stem cells and in early embryogenesis, but its expression is downregulated with differentiation (Moss and Tang, 2003). Recently, Lin28 was also used with three other factors (OCT4, SOX2, NANOG) to reprogramme human somatic fibroblasts to pluripotency (Yu *et al*, 2007). This finding suggests that Lin28 is related to stem cell function.

On the other hand, it was recently reported that Lin28 is upregulated in human tumours and functions as an oncogene promoting transformation and tumour progression (Viswanathan *et al*, 2009). Depletion of Lin28 and expression of let-7 suppressed bone metastasis, while Lin28 expression resulted in bone metastasis in mice implanted with breast tumour cells (Dangi-Garimella *et al*, 2009). Lin28B protein, a homologue of Lin28, is also overexpressed in hepatocellular carcinoma and induction of expression with exogenous Lin28B promoted cancer cell proliferation (Guo *et al*, 2006). Lin28B is also induced by Myc and plays an important role of Myc-dependent cellular proliferation (Chang *et al*, 2009). Thus, altered expression of Lin28 seems to promote tumourigenesis and can be associated with advanced malignancy.

In this study, we analysed the expression of Lin28 and Lin28B, a homologue of Lin28, in oesophageal cancer by immunohistochemistry and determined the relationship between their expression and various clinicopathological parameters including prognosis of patients. Moreover, in *in vitro* studies, we also examined the relationship between Lin28B expression and aggressiveness of oesophageal cancer cells using oesophageal cell lines.

## Patients and Methods

### Patients and tissue samples

All 161 tissue samples were obtained from patients who underwent radical oesophagectomy with lymph node dissection for thoracic oesophageal cancers between 2000 and 2006 at the Department of Gastroenterological Surgery, Graduate School of Medicine, Osaka University. Informed consent was obtained from each patient at that of surgery. Of these patients, 94 received preoperative chemotherapy followed by surgery while the remaining 67 patients underwent surgery without preoperative therapy. The preoperative chemotherapeutic regimen was cisplatin at 70 mg m^–2^, adriamycin at 35 mg m^–2^ (by rapid intravenous infusion on day 1), and 5-FU at 700 mg m^–2^ (by continuous intravenous infusion on day 1 through day 7) (Miyata *et al*, 2009). Two courses of chemotherapy were used, separated by a 4-week interval. The median duration of follow-up was 32.7 months (range, 3.1–97.9 months), and 72 patients (44.7%) died during the follow-up period.

### Immunohistochemistry

A staining score of Lin28 and Lin28B was calculated based on the proportion of immunostained cancer cells to that of all cancer cells in three fields of view. The expression level was categorised as high (staining score >50%) or low (staining score ⩽50%).

For immunohistochemistry, 4 *μ*m-thick sections cut from formalin-fixed, paraffin-embedded (FFPE) tissue blocks were deparaffinised and rehydrated using xylene and serial dilutions of ethanol. Antigen retrieval was performed in 1 mmol l^–1^ sodium citrate buffer (pH 6.0 for Lin28, pH9.0 for Lin28B) by autoclave treatment at 121°C for 15 min, and then the sections were incubated with goat serum for 20 min to block nonspecific binding followed by incubation with the primary polyclonal rabbit antibody; anti-Lin28 (1 : 200 dilution; Proteintech, Chicago, IL, USA) at room temperature for 2 h and anti-Lin28B (1 : 50 dilution; Cell Signaling, Danvers, MA, USA) overnight at 4°C. After incubation with anti-rabbit secondary antibody (Vector Laboratories, Burlingame, CA, USA) for 20 min, the antigen–antibody complexes were visualised using VECTASTAIN ABC kit (Vector Laboratories) according to the protocol supplied by the manufacturer. The sections were counterstained with haematoxylin. All sections were examined independently by two coauthors (R Hamano and H Miyata), who were blinded to the clinical information.

### Clinical and histopathological evaluation of response to chemotherapy

The clinical response to chemotherapy was categorised according to the criteria of the World Health Organization response criteria for measurable disease (Miller *et al*, 1981) and the Japanese Society for Esophageal Diseases (Japan Esophageal Society, 2009b). A complete response was defined as total regression of the primary tumour and its disappearance on CT scan and/or PET scan and endoscopy. A partial response (PR) was defined as >50% reduction in primary tumour size and lymph node metastasis, as confirmed by CT scan. Progressive disease (PD) was defined as >25% increase in the size of the primary tumour or the appearance of new lesions. Cases that did not meet the criteria of PR or PD were defined as stable disease.

For histopathological assessment, serial 4 *μ*m-thick tissue sections of the primary tumour and lymph nodes were cut from the surgical specimens, fixed with 10% buffered formalin, embedded in paraffin, and stained with haematoxylin and eosin. The extent of viable residual carcinoma at the primary site was assessed semiquantitatively, based on the estimated percentage of viable residual carcinoma in relation to the macroscopically identifiable tumour bed that was evaluated histopathologically. Briefly, the percentage of viable residual cancer cells within the total cancerous tissue was assessed as follows: grade 3, no viable residual tumour cells; grade 2, <2/3 residual tumour cells; grade 1b, 1/3–2/3 residual tumour cells; grade 1a, >2/3 residual tumour cells; grade 0, no significant response to chemotherapy (Miyata *et al*, 2009; Japan Esophageal Society, 2009a).

### Cell lines and culture conditions

Five established cell lines derived from oesophageal squamous cell carcinoma (TE-1, -8, -10, -13, -15) were obtained from the Riken Cell Bank (Tsukuba, Japan). All cell lines were cultured in RPMI 1640 (Life Technologies, Gaithersburg, MD, USA) containing 10% fetal bovine serum (Sigma-Aldrich Co., St Louis, MO, USA) and 1% penicillin/streptomycin (Life Technologies Inc.), under a humidified atmosphere with 5% CO_2_ at 37°C.

### Immunoblotting

Adherent cells grown to 50–80% confluence were washed with ice-cold phosphate-buffered saline and lysed in RIPA buffer (Thermo Fisher Scientific Inc., Waltham, MA, USA) or Sample buffer (Wako Pure Chemical Industries, Osaka, Japan) and a cocktail of phosphatase inhibitors (Thermo Fisher Scientific Inc.) on ice. Lysates were spun and the supernatant was collected. Equal amounts of cell extracts (15 *μ*g) were fractionated by SDS–PAGE gel (Bio-Rad Laboratories, Hercules, CA, USA) and transferred onto hydrophobic polyvinylidene difluoride membranes (ImmobilonP, Millipore, Bedford, MA, USA). The membranes were blocked by incubation in 5% milk followed by incubation overnight at 4°C with the primary antibodies, and then with the secondary antibodies for 1 h at room temperature. The following antibodies were used in this study; anti-actin (dilution, 1 : 1000, Sigma-Aldrich Co.), and anti-Lin28B (dilution, 1 : 1000, Abcam, Cambridge, MA, USA). Immune complexes were detected using Detection Kit (GE Healthcare, Buckinghamshire, UK).

### Small interfering RNA transfection

Cells were cultured to 60–80% confluence and transfected with 1 *μ*M of small interfering RNAs (siRNAs) that targeted human Lin28B (Si-Lin28B) or negative control oligonucleotides (Applied Biosystems, Foster City, CA, USA) using siPORT NeoFX Transfection Agent (Ambion, Austin, TX, USA) according to the protocol provided by the manufacturer. After transfection, the cells were cultured for 72 h and intermediate samples were collected at 24 and 48 h and analysed by immunoblotting and MTT (3-(4,5-dimethylthiazol-2-yl)-2,5-diphenyltetrazolium bromide) assay.

### MTT cell proliferation assay

The MTT assay was used to assess proliferative activity. After siRNA transfection, cells were seeded into 96-well plates at 5 × 10^3^ per well and incubated overnight under standard culture condition. Following incubation for 24, 48, 72, and 96 h, 10 *μ*l of MTT solution was added to each well and the plates were incubated for another 3 h at 37°C, and formazan crystals were dissolved with 100 *μ*l of 0.04 N HCl-isopropanol. The absorbance of individual wells was read at 550 nm test wavelength and 655 nm reference wavelengths using a microplate reader (Bio-Rad Laboratories). Cell proliferation activities of siRNA transfected cells and negative control transfected cells were determined by absorbance values.

### Invasion assay

*In vitro* cell invasion was assayed using the BioCoat Matrigel Invasion Chambers (Becton Dickinson Biosciences, Sparks, MD, USA) using the procedure recommended by the manufacturer. Briefly, the transfected cells were harvested and placed in the upper chamber (2.5 × 10^5^ cells per well) in serum-free medium. After incubation at 37°C for 48 h to allow invasion of the Matrigel-coated chamber, the invaded cells on the lower surface were fixed and stained using Diff-Quik stain kit (Dade Behring Inc., Newark, DE, USA), whereas the noninvading cells on the upper surface were scraped and washed away. Finally, the number of invaded cells was counted under a microscope in nine random fields ( × 200).

### RNA isolation from FFPE specimens

Total RNA was isolated from the FFPE tissue specimens using the RecoverAll Total Nucleic Acid Isolation Kit (Ambion) according to the instructions supplied by the manufacturer. Briefly, each FFPE tissue block was cut into 20-*μ*m thick pieces, and four slices were placed in a centrifuge tube. To liquefy the paraffin, 100% xylene and 100% ethanol was added to each tube. After centrifugation, the precipitated samples were air dried and treated with protease in heat blocks for 3 h at 50°C. Then, each sample was treated with isolation reagent and filtered. Each filter was treated with DNase and incubated for 30 min at room temperature. After washing the filter with washing reagents, it was treated with warmed Elution Solution and centrifuged to pass the mixture through the filter. The eluate contained the isolated RNA.

### Quantitative real-time reverse transcription–PCR

The complementary DNA was synthesised from 10 ng of total RNA using the TaqMan miRNA Reverse Transcription Kit and specific stem-loop reverse transcription primers (Applied Biosystems) according to the protocol provided by the manufacturer. The reverse transcription was set at these conditions: 16°C for 30 min followed by 40°C for 30 min and 85°C for 5 min. Real-time PCR reaction was performed using TaqMan Universal PCR master mix No AmpErase UNG and TaqMan miRNA specific PCR primers (Applied Biosystems). A 20 *μ*l of the reaction product was incubated in a 96-well optical plate at 95°C for 10 min, followed by 40 cycles at 95°C for 15 s, and 60°C at 1 min using ABI PRISM 7900HT (Applied Biosystems). The miRNA expression value was expressed relative to that of RNU48 and analysed using the 2^−ΔΔCt^ method (Livak and Schmittgen, 2001; Hamano *et al*, 2011).

### Statistical analysis

All data are expressed as mean±s.d. The relationship between miRNA expression and each clinicopathological variable was analysed by *χ*^2^ test, Fisher's exact test, or Mann–Whitney *U*-test. Time to recurrence was defined as the time interval between the date of surgery and the date of diagnosis of first recurrence or last date of follow-up if recurrence was not observed. Overall survival time was censored at the date of the last follow-up if death did not occur. For survival analysis, the Kaplan–Meier method was used to assess survival time distribution according to miRNA expression level and the log-rank test was used to examine the differences between groups. A *P*-value of <0.05 denoted the presence of statistically significant difference between groups. All statistical analyses were performed with JMP ver. 8.0 software (SAS Institute Inc., Cary, NC, USA).

## Results

### High expression of Lin28 and Lin28B in oesophageal cancer cells

**Figure 1:**
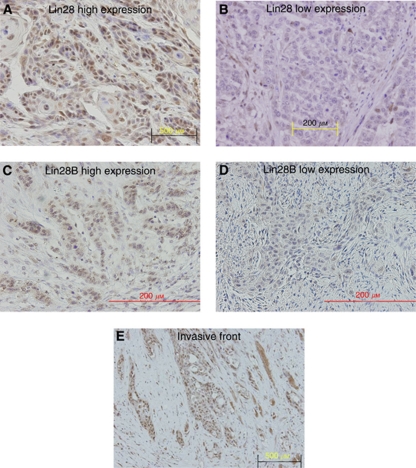
Lin28 and Lin28B immunostaining in oesophageal cancer. (**A**) Lin28 positively stained cells. (**B**) Lin28 negatively stained cells. (**C**) Lin28B positively stained cells. (**D**) Lin28B negatively stained cells. (**E**) The carcinoma cells at the invasive front.

First, we determined the expression pattern of Lin28 and Lin28B in cancerous and non-cancerous tissues from patients with oesophageal cancer. Lin28 staining was predominantly detected in the nuclei of cancer cells, though cytoplasmic staining was also evident in some cancer cells (Figure 1A and B). Lin28B staining was also predominantly detected in the nuclei of cancer cells (Figure 1C and D). This finding is in agreement with the results of a previous study of Lin28 expression in the cytoplasm and its transportation to the nucleus (Balzer and Moss, 2007). The carcinoma cells at the invasive front tended show strong staining for Lin28 and Lin28B compared with those in other areas (Figure 1E). On the other hand, in non-cancerous tissue, differentiated cells were not stained although the cytoplasm of some basal cells was stained weakly for Lin28 and Lin28B.

### High expression of Lin28 is associated with poor prognosis

Tables 1 and 2 summarise the relationship between Lin28 and Lin28B expression and various clinicopathological parameters. There were no significant relationships between Lin28 expression and tumour differentiation or tumour depth. However, high expression of Lin28 correlated significantly with lymph node metastasis (*P*=0.035) and lymphatic vessel invasion (*P*=0.047). Similar to Lin28, high expression of Lin28B correlated significantly with lymph node metastasis (*P*=0.017) and lymphatic vessel invasion (*P*=0.002). Moreover, the expression of Lin28B significantly correlated with tumour depth (*P*=0.005).

**Figure 2:**
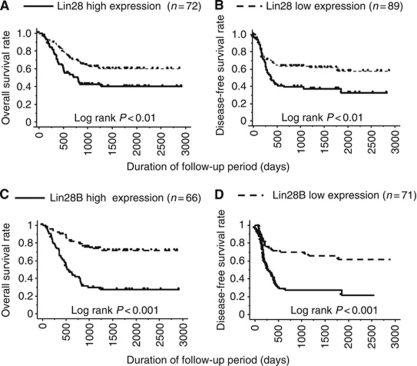
Correlation between Lin28/Lin28B expression and survival of 161 patients with oesophageal cancer. High expression of Lin 28 (**A** and **B**)/Lin28B (**C** and **D**) correlated with shortened survivals.

High expression of Lin28 correlated significantly with shortened survival including both overall survival and disease-free survival (Figure 2A and B). High expression of Lin28B also correlated significantly with shortened survival (Figure 2C and D). Furthermore, multivariate analysis identified Lin28B expression as an independent prognostic factor, along with the number of metastatic lymph nodes (Table 3). These results suggest that Lin28 and Lin28B may influence the malignant potential of oesophageal cancer. However, Lin28 expression did not correlate with clinical and pathological responses to preoperative chemotherapy (Table 4).

### Inhibition of Lin28B expression regulates cellular behaviour

**Figure 3:**
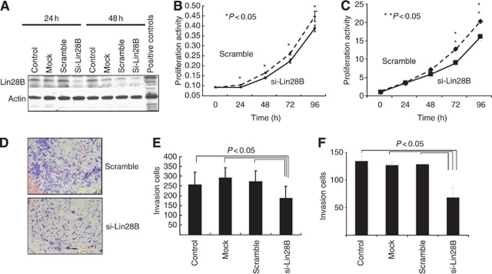
Proliferative and invasive activities of Lin28B-knockdown cells. (**A**) Western blotting to confirm reduced Lin28B expression following transfection of si-Lin28B in TE-13. (**B**) Proliferative activities of Lin28B-knockdown cells and control cells in TE-13. (**C**) Proliferative activities of Lin28B-knockdown cells and control cells in TE-10. (**D**) Invasive activities of Lin28B-knockdown cells and control cells in TE-13. (**E**) Quantitative analysis of invasive activity in TE-13 (data are mean±s.d. of three experiments). (**F**) Quantitative analysis of invasive activity in TE-10 (data are mean±s.d. of three experiments).

The above results suggest that Lin28 and Lin28B are potentially associated with aggressiveness of oesophageal cancer. In the next series of studies, *in vitro* experiments were conducted to examine the effect of these expressions on the malignant potential of oesophageal cancer cells. First, we screened several oesophageal cancer cell lines, and found that some cell lines express Lin28B, while expression of Lin28 is quite low in all cell lines examined (data not shown). Thus, to study the effects of expression of Lin28B on cellular proliferation, its expression was knocked down by transfecting si-Lin28B in TE-13 oesophageal cancer cells (Figure 3A). The proliferative activity of Lin28B-knockdown cells was significantly reduced compared with that of control cells (Figure 3B). Second, the invasion assay was conducted to assess the role of Lin28B in lymph node metastasis by invasion to lymphatics. The invasive activity of Lin28B-knockdown cells was clearly reduced compared with that of negative control cells (Figure 3D and E). In another oesophageal cancer cell line, TE-10, the reduced proliferation and invasive activity of Lin28B-knockdown cells were confirmed (Figure 3C and F).

### Relationship between Lin28 expression and let-7 expression

**Figure 4:**
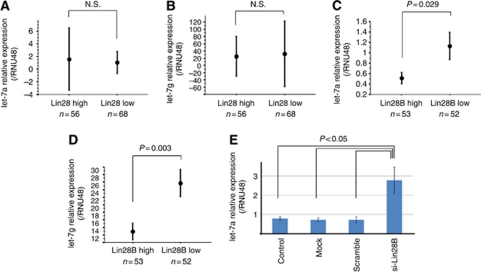
Relationship between Lin28B expression and let-7 expression in oesophageal cancer. (**A** and **B**) Analysis of surgical specimens showed no significant relationship between Lin28 expression and let-7 expression, determined by real-time RT–PCR. (**C** and **D**) Analysis of surgical specimens showed significantly relationship between Lin28B expression and let-7 expression, determined by real-time RT–PCR. (**E**) *In vitro* assay using oesophageal cancer cell showed upregulation of let-7 expression in Lin28B-knockdown cells, compared with control cells.

Lin28 is described as a negative regulator of let-7 biogenesis (Heo *et al*, 2008; Viswanathan *et al*, 2008). We investigated the relationship between expression of Lin28 and Lin28B and let-7 expression. We found significant relationship between let-7 expression and Lin28B expression, but not Lin28 expression in the surgical specimens of oesophageal cancer (Figure 4A–D). *In vitro* assay showed that let-7 was upregulated in cultured Lin28B-knockdown oesophageal cancer cells, compared with control cells (Figure 4E). This finding is consistent with the results of the previous study showing a relationship between Lin28B and let-7 (King *et al*, 2011).

## Discussion

Lin28 is a negative regulator of let-7 family, which may act as a tumour suppressor miRNA, suggesting that Lin28 could contribute to tumourigenesis. The present study demonstrated that high expression of Lin28 and Lin28B is associated with lymph node metastasis and poor prognosis of patients with oesophageal cancers. *In vitro* studies confirmed that Lin28B expression was associated with aggressiveness of oesophageal cancer through increased proliferation and invasive activities in oesophageal cancer cells.

Recent studies suggest that Lin28 functions as an oncogene promoting malignant transformation and tumour progression (Viswanathan *et al*, 2009). Indeed, several recent reports demonstrated that Lin28 expression correlates with survival of patients with malignant diseases (Guo *et al*, 2006). In ovarian cancer, patients with high Lin28B expression had shorter progression-free and overall survival times than those with low Lin28B expression (Lu *et al*, 2009). In another recent report, high Lin28B staining intensity in stage I/II colon cancers correlated with reduced survival and increased probability of tumour recurrence (King *et al*, 2011). Our result of the correlation between high expression of Lin28 and Lin28B and poor prognosis of patients with oesophageal cancers is compatible with the above studies. Thus, Lin28 expression may be clinically relevant prognostic marker in various malignancies including oesophageal cancer.

The present study demonstrated that Lin28 expression is associated with tumour aggressiveness through increased proliferation of oesophageal cancer cells. One recent study demonstrated that Lin28B is necessary and sufficient for Myc-mediated let-7 repression, and that Lin28B has an important role in Myc-dependent cellular proliferation (Chang *et al*, 2009). Another study showed that high expression of Lin28 and Lin28B correlated with low let-7 expression and upregulation of let-7 target such as HMGA2 and Myc, and that knockdown of Lin28B expression impaired cellular proliferation (Viswanathan *et al*, 2009). Thus, Lin28 may increase proliferation of oesophageal cancer cells by directly inhibiting let-7 expression and subsequently upregulating HMGA2 and Myc, which are targets of let-7.

In this study, high expression of Lin28 correlated significantly with lymph node metastasis in patients with oesophageal cancers. A recent study showed that Raf kinase inhibitory protein repressed breast tumour cell intravasation and bone metastasis in a mouse model, through inhibition of mitogen-activated protein kinase, leading to decreased transcription of Lin28 and enhanced expression of let-7 by Myc (Dangi-Garimella *et al*, 2009). These data provided the first evidence for the roles of Lin28 and let-7 expression in tumour metastasis, in addition to the regulation of tumour growth. Another recent study demonstrated that constitutive expression of Lin28B expression in colon cancer cells confers metastatic ability by showing that mice xenografted with Lin28B expressing colon cancer cells developed much more metastasis in the liver, lung, and mesenterium compared with mice of the empty vector control group (King *et al*, 2011). Thus, Lin28 expression may have an important role in cancer metastasis. In the present study, Lin28B expression was significantly associated with the invasive activity of oesophageal cancer cells. The result that high expression of Lin28 correlated with lymph node metastasis in oesophageal cancer may depend on the increased invasiveness through Lin28 expression.

Several recent studies have identified the important roles of Nanog, Sox2 and Oct3/4, which are involved in reprogramming and maintenance of stem cell function, in tumour aggressiveness (Ben-Porath *et al*, 2008). Immunohistochemical analysis of gastric cancer (Lin *et al*, 2011) and colorectal cancer (Meng *et al*, 2010) showed that overexpression of Nanog correlated strongly with lymph node metastasis and poor prognosis of patients. In hepatocellular carcinoma, Sox2 and Oct4 were identified as independent prognostic factors with poorest prognosis in patients with tumours that co-expressed Sox2/Oct4 proteins (Huang *et al*, 2011). In oesophageal cancer, the expression of Oct3/4 and Sox2 proteins was reported to correlate with advanced cancer, which in turn correlated with poor clinical outcome (Wang *et al*, 2009). In our study, Lin28, a reprogramming factor, was associated with tumour aggressiveness and poor prognosis of patients with oesophageal cancer. Thus, reprogramming factors, which regulates stem cell like properties such as pluripotency and self-renewal in normal cells, may confer the high malignant potential of cancer cells.

In the present study, Lin28B expression correlated inversely with let-7 expression in oesophageal cancer cell line and human oesophageal cancers, although no such correlation was identified between Lin28 and let-7 expression. Several studies have indicated that Lin28/Lin28B is part of the regulatory network that also involves let-7. Lin28/Lin28B represses let-7, which itself represses Lin28/Lin28B by binding to the 3′UTR of Lin28/Lin28B transcripts, thus forming a double-negative feedback loop. A second feedback loop is that Lin28/Lin28B de-represses c-Myc by inhibiting let-7, and c-Myc transcriptionally activates Lin28/Lin28B (Chang *et al*, 2009; Dangi-Garimella *et al*, 2009). A third feedback loop involves NF-*κ*B, Lin28B, let-7 and IL-6 (Iliopoulos *et al*, 2009). NF-*κ*B induces Lin28B expression, leading to inhibition of let-7 and expression of the encoding IL-6 (a let-7 target). IL-6 can itself activate NF-*κ*B, resulting in a positive feedback loop. Thus, Lin28 and let-7 may form a complex feedback loop in malignant transformation. Moreover, one recent study showed that Lin28 and Lin28B function through distinct mechanisms to block let-7 processing (Piskounova *et al*, 2011). Further studies are required to elucidate the roles of Lin28/Lin28B and let-7 network in oesophageal cancers.

In summary, we examined in the present study the clinical significance of Lin28 and Lin28B expression in oesophageal cancer and demonstrated that high expressions of Lin28 and Lin28B are associated with lymph node metastasis and poor prognosis of patients with oesophageal cancers. Moreover, *in vitro* studies confirmed that Lin28B expression was associated with aggressiveness of oesophageal cancer by increasing the proliferation and invasiveness of oesophageal cancer cells. Further studies are needed to confirm the role of feedback loops including Lin28/Lin28B and let-7 in oesophageal cancer.

## Figures and Tables

**Table 1:** Correlation between Lin28 expression and various clinicopathological features of patients with oesophageal cancer

	**Lin28 expression**		
	**High**	**Low**	***P*-value**
*n*	72	89	
*Sex*			
Male/female	63/9	81/8	0.607
			
*Age (years)*			
Mean±s.d.	61.99±9.5	64.23±7.7	0.101
			
*pStage*			
0	0	2	0.068
1	8	16	
2	21	26	
3	19	26	
4	24	19	
			
*Histology*			
Well SCC	18	14	0.413
Moderate SCC	32	49	
Poor SCC	15	16	
Other	7	10	
			
*pT*			
0	0	2	0.249
1	14	24	
2	13	11	
3	35	45	
4	10	7	
			
*pN*			
0	19	38	0.046
1	53	51	
Mean±s.d.	6.68±16.9	2.73±4.3	0.035
			
*pM*			
0	47	70	0.042
1	3	0	
1a	5	9	
1b	17	10	
			
*Lymphatic invasion*			
+	63	66	0.047
−	9	23	
			
*Venous invasion*			
+	34	42	0.999
−	38	47	
			
*Preoperative chemotherapy*			
+	46	48	0.260
−	26	41	
			
*Curative surgery*			
Yes	64	85	0.137
No	8	4	

Abbreviation: SCC=squamous cell carcinoma.

**Table 2:** Correlation between Lin28B expression and various clinicopathological features of patients with oesophageal cancer

	**Lin28B expression**		
	**High**	**Low**	***P*-value**
*n*	66	71	
*Sex*			
Male/female	57/9	65/6	0.416
			
*Age (years)*			
Mean±s.d.	62.3±9.5	62.8±7.7	0.730
			
*pStage*			
0	0	2	0.003
1	3	13	
2	17	21	
3	23	21	
4	23	14	
			
*Histology*			
Well SCC	15	12	0.358
Moderate SCC	29	42	
Poor SCC	15	11	
Other	7	6	
			
*pT*			
0	0	2	0.005
1	6	21	
2	14	7	
3	34	38	
4	12	3	
			
*pN*			
0	14	29	0.017
1	52	42	
Mean±s.d.	5.17±9.2	4.54±15.3	0.773
			
*pM*			
0	44	55	0.135
1	1	0	
1a	7	6	
1b	14	10	
			
*Lymphatic invasion*			
+	61	50	0.002
−	5	20	
			
*Venous invasion*			
+	36	28	0.088
−	30	43	
			
*Preoperative chemotherapy*			
+	45	39	0.119
−	21	32	
			
*Curative surgery*			
Yes	56	70	0.004
No	10	1	

Abbreviation: SCC=squamous cell carcinoma.

**Table 3:** Results of univariate and multivariate Cox's models for disease-free survival

			**Univariate analysis**		**Multivariate analysis**	
	***n***	**%**	**HR (95% CI)**	***P*-value**	**HR (95% CI)**	***P*-value**
*Sex*						
Males	144	89.4	0.986 (0.380–2.563)	0.9774		
Females	17	10.6				
						
*Age*						
>65 years	76	47.2	1.616 (0.469–1.560)	0.6104		
⩽65 years	85	52.8				
						
*pT*						
3–4	97	60.2	10.354 (0.193–0.680)	0.0013	1.242 (0.350–1.854)	0.6105
0–2	64	39.8				
						
*pN*						
1	104	64.6	30.630 (0.073–0.311)	<0.001	4.310 (0.094–0.570)	0.001
0	57	35.4				
						
*Differentiation*						
Other	129	80.1	1.976 (0.271–1.249)	0.1599		
Well	32	19.9				
						
*Curability*						
R1–2	12	7.5	3.994 (1.013–1.072)	0.0457	1.319 (0.140–4.109)	0.7484
R0	149	92.5				
						
*Lin28B expression*						
Low	71	51.8	0.166 (0.079–0.349)	<0.001	0.193 (0.087–0.432)	<0.001
High	66	48.2				

Abbreviations: HR=hazard ratio; 95% CI=95% confidence interval.

**Table 4:** Relationship between Lin28 expression and response to preoperative chemotherapy in patients with oesophageal cancer

	**Lin28 expression**			**Lin28B expression**		
	**High**	**Low**		**High**	**Low**	
	***n*=46**	***n*=48**	***P*-value**	***n*=45**	***n*=39**	***P*-value**
*Clinical response*						
CR	0	3	0.621	1	2	0.126
PR	24	22		19	21	
SD–PD	22	23		25	16	
						
*Pathological response*						
2	3	6	0.210	1	4	0.103
1b	8	11		7	10	
1a	20	19		22	15	
0	15	12		15	10	

Abbreviations: SD=stable disease; PD=progressive disease; PR=partial response; CR=complete response.
